# Perioperative Management of a Frail Patient With Bernard-Soulier Syndrome

**DOI:** 10.7759/cureus.53546

**Published:** 2024-02-04

**Authors:** Pedro Goncalves, Magna Fortunato

**Affiliations:** 1 Anesthesiology, Centro Hospitalar Universitário São João, Porto, PRT

**Keywords:** anesthesia, antifibrinolytics, recombinant activated factor vii, perioperative blood management, bernard-soulier syndrome

## Abstract

Bernard-Soulier syndrome (BSS) is an autosomal recessive inherited bleeding disorder characterized by prolonged bleeding time, thrombocytopenia, and giant platelets. Patients with BSS are at an increased risk of bleeding, especially during traumatic injury and surgical procedures. The literature on the anesthetic management of patients with BSS is limited. In this report, we detail the successful management of a frail patient with BSS who underwent a major surgical procedure. Despite comprehensive clinical monitoring and an extended pharmacological strategy, a hemorrhagic complication occurred in the later postoperative phase, emphasizing the necessity for continued support and vigilant clinical monitoring due to the ongoing bleeding risk associated with these patients. In this case, a combined strategy involving antifibrinolytics, recombinant factor VII, and platelet transfusions proved effective.

## Introduction

Bernard-Soulier syndrome (BSS) is a rare autosomal recessive bleeding disorder resulting from a deficiency or dysfunction of the glycoprotein Ib-IX-V (GPIb-IX-V) complex on the platelet surface. This complex is crucial for von Willebrand factor (vWF) binding and platelet adhesion. Patients with BSS are at a heightened risk of bleeding, particularly during traumatic injury and surgical procedures [1,2]. Managing regional anesthesia in these cases may be challenging due to increased bleeding risks. This report details the anesthetic approach applied to a 75-year-old patient with BSS undergoing a laparoscopic right hemicolectomy. The literature regarding anesthetic management in such patients is limited, and as far as we know, this case represents the first documented case in an elderly patient.

## Case presentation

A 75-year-old female patient underwent elective right hemicolectomy due to a large colon neoplastic polyp. While she had a history of menometrorrhagias and an abnormal postpartum hemorrhage leading to hysterectomy, the BSS diagnosis was made at 55 years of age following routine laboratory screening that revealed mild thrombocytopenia. She had no other significant medical history, no family history of bleeding disorders, and no further personal bleeding episodes after diagnosis. At 73 years of age, the patient underwent a colonoscopy with polypectomy, requiring the administration of one unit of platelet concentrate but with no complications, hemorrhagic or otherwise. Since her diagnosis, this was the only invasive procedure the patient was subjected to.

**Figure 1:**
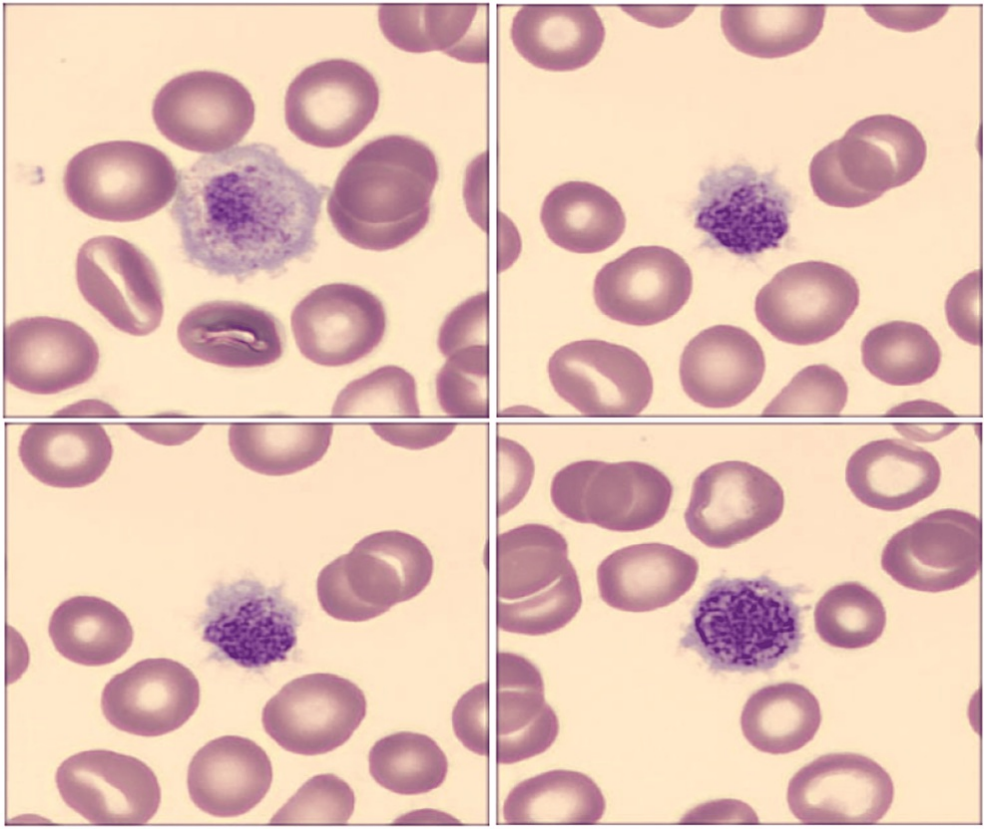
Abnormally large and irregularly shaped platelets seen in the preoperative microscopic examination Peripheral blood smear was carried out with Sysmex SP-50 and the photograph digitized with Sysmex DI-60 (Sysmex, Kobe, Japan).

Preoperative investigations showed a hemoglobin level of 13 g/dL, platelet count of 30 x 10^9^/L (normal range: 150-450 x 10^9^/L), a normal coagulation profile, and an abnormal peripheral blood smear displaying abnormally large and irregularly shaped platelets (Figure 1). The high bleeding risk required multidisciplinary collaboration among immunohematology specialists, surgeons, and anesthesiologists.

The strategy outlined for this case involved a combined approach of antifibrinolytics, platelet transfusions, and the use of recombinant factor VII (rFVII), beginning in the preoperative period. Consequently, the patient received a single unit of apheresis platelets one hour pre-surgery.

After obtaining informed consent and initiating standard monitoring, a radial arterial line was inserted to continuously monitor blood pressure and perform arterial blood gas analysis. Additionally, anesthetic depth was measured with bispectral index (BIS) and acceleromyographic train of four (TOF) was employed to gauge the extent of neuromuscular blockade. The induction of anesthesia involved administering fentanyl 100 mcg IV, propofol at a dosage of 2 mg/kg, and rocuronium at 1.2 mg/kg. Sevoflurane was adjusted to sustain a BIS within the range of 40-60 for anesthesia maintenance. To ensure sufficient neuromuscular blockade, a rocuronium infusion was maintained at a rate of 0.1 mg/kg/h.

Preceding the surgical incision, a single dose of rFVIIa (6 mg) and 1 g of tranexamic acid (TXA) were administered. Another dose of rFVIIa (6 mg) was given intraoperatively after two hours. The surgery lasted 2.5 hours, maintaining the patient's hemodynamic stability without requiring vasopressor support. Approximately 200 ml of blood loss occurred, and fluid balance was upheld with 400 ml of balanced crystalloid solution. For increased monitoring of blood losses, a drain was placed in the right paracolic gutter.

Following the surgery, a multimodal analgesia regimen comprising 1000 mg of acetaminophen, 100 mg of tramadol, and 5 mg of morphine was provided. Reversal of neuromuscular blockade was achieved using 2 mg/kg of sugammadex, facilitating safe extubation once the TOF ratio exceeded 0.9. The patient was transferred to the post-anesthesia care unit (PACU), awake, breathing spontaneously without the need for oxygen support, hemodynamically stable, and comfortable, reporting a visual analogue scale (VAS) score of zero. The immediate postoperative hemoglobin was 13 g/dL, and the platelet count was 39 x 10^9^/L. The coagulation profile, lactate levels, and the rest of the blood gas analysis during the procedure were within normal ranges.

Postoperatively, the patient remained in the PACU where she was scheduled to receive 6 mg of rFVIIa four hours after the end of surgery and another 6 mg of rFVIIa six hours after that. Another unit of apheresis platelets was given in the immediate postoperative period followed by another apheresis unit six hours after that.

On the subsequent postoperative day, she received TXA every eight hours and underwent apheresis platelet transfusions twice daily. By the second postoperative day, the patient remained stable without any clinically concerning bleeding, allowing for a reduction in TXA to twice daily and apheresis platelet transfusions to one unit daily. Her postoperative recovery progressed uneventfully, leading to PACU discharge and transfer to the inpatient ward on the third postoperative day. TXA every 12 hours and daily apheresis platelet units were continued in the following days.

On the ninth postoperative day, following a platelet transfusion, the drain was removed. No additional allogeneic blood products were administered throughout the rest of the postoperative course. Despite her platelet count decreasing to a nadir of 15,000/mm^3^, there were no clinically concerning bleeding issues, leading to her discharge from the hospital on the 10th postoperative day.

Forty-eight hours after her discharge, she returned to the emergency department due to a series of hematochezia episodes. Her vital signs were normal, and her blood tests indicated a hemoglobin level of 10.3 g/dL and a platelet count of 57 x 10^9^/L. Another unit of platelet transfusion was administered, and she was then transferred to the surgical ward for further care. Due to ongoing hematochezia, her hemoglobin level dropped to 5.1 g/dL. Despite receiving support and ongoing transfusions, she developed hemorrhagic shock, necessitating admission to the intensive care unit.

A massive transfusion protocol was initiated, and a contrast computed tomography (CT) scan was conducted, confirming no active gastrointestinal bleeding. After resuscitation and stabilization, she resumed the intravenous TXA protocol every 12 hours and received a daily unit of apheresis platelets, which continued for another 10 days. She was discharged from the hospital 48 hours later, on the 12th day, in a stable condition.

## Discussion

BSS typically manifests as an autosomal recessive disorder, often diagnosed in childhood. However, milder forms with an autosomal dominant pattern may emerge later in life. The primary genetic mutations affect genes responsible for the platelet surface glycoprotein complex, particularly glycoprotein Ib-IX-V [1,2]. This complex's role in platelet adhesion, binding to von Willebrand factor and exposed collagen in damaged vessels, becomes impaired in BSS. Physiopathologically, the reduced binding affinity between the mutated GPIb-IX-V complex and vWF/collagen impairs platelet adhesion, leading to decreased platelet aggregation, prolonged bleeding time, and heightened susceptibility to spontaneous bleeding episodes. Platelet counts typically fluctuate between 20 and 100 × 10^9^/L [2].

Frailty potentially heightens the risk of bleeding complications in individuals with BSS. The multifaceted nature of frailty, encompassing reduced physiological reserves, altered immune responses, and heightened inflammatory processes, intersects with the coagulation system, impacting its delicate equilibrium and increasing vulnerability to bleeding or clotting issues.

Furthermore, the presence of comorbidities, prevalent among elderly and frail populations, further influences coagulation dynamics. Conditions such as cardiovascular disease, diabetes, chronic kidney disease, and malignancies intricately interweave with coagulation processes, potentially exacerbating thrombotic or hemorrhagic events.

Frail individuals may exhibit weaker blood vessels, compromised tissue integrity, and a diminished capacity to respond to or recover from bleeding episodes. A decline in the reserve of hematopoietic stem cells as people age might lead to reduced platelet production in elderly individuals. Simultaneously, there tends to be an increase in platelet reactivity. While aging is well-established as being associated with hypercoagulability, the evidence regarding specific coagulation changes specifically due to frailty remains less clear. Studies focusing on individual factors in the coagulation system suggest that frailty is associated with pro-coagulant changes [3,4].

Therapeutic options available for reducing hemorrhagic risk

Recombinant Factor VIIa

As part of the extrinsic pathway, factor VII (FVII) holds a crucial role in initiating the coagulation cascade upon vascular injury or tissue damage. Damaged tissues release tissue factor (TF), also referred to as factor III, which forms a complex with circulating FVII, resulting in the activation of FVII to its active form, FVIIa. This TF-FVIIa complex plays a fundamental role in triggering the coagulation cascade. Activated FVIIa then interacts with factor X (FX) and its cofactor, factor Va, in the presence of calcium ions and phospholipids on cell membranes. This interaction forms the prothrombinase complex, efficiently converting prothrombin (factor II) into thrombin (factor IIa). Recombinant factor VIIa is a synthetic form of this factor used in various bleeding disorders. Despite BSS primarily involving platelet dysfunction, rFVIIa has been considered as an adjunctive treatment in severe bleeding episodes. It aids in enhancing blood clot stability by promoting the cross-linking of fibrin. However, the use of rFVIIa in BSS is limited and not a standard treatment due to its potential to promote thrombosis. Hence, careful monitoring and judicious administration are necessary [5,6].

Antifibrinolytics

Tranexamic acid, an antifibrinolytic agent, inhibits blood clot breakdown by reversibly blocking plasminogen's lysine binding sites. It prevents plasminogen conversion to plasmin, thereby halting fibrin degradation. This agent has been investigated as an adjunctive treatment in BSS to reduce bleeding episodes, particularly when platelet transfusions are ineffective or unavailable. Optimizing dosage and timing requires careful consideration to balance its antifibrinolytic effects with thrombosis risk [7].

Aminocaproic acid is another antifibrinolytic agent that has shown efficacy in managing bleeding disorders, including during surgeries, trauma, and certain medical conditions. Studies have demonstrated its ability to effectively reduce bleeding and transfusion requirements. The key differences lie in their pharmacokinetics and dosing. TXA generally has a longer duration of action, allowing for less frequent dosing compared to aminocaproic acid. However, TXA is better tolerated and 10-fold more potent than aminocaproic acid in vitro [7].

Desmopressin

Desmopressin (DDAVP), capable of stimulating vWF release and temporarily enhancing platelet adhesion, is at times utilized alongside platelet transfusions in managing hemorrhagic shock in BSS. However, its efficacy in severe bleeding situations may be limited. The effects of desmopressin are relatively short-lived. This means that it may not provide a sustained response in situations where continuous clotting factor support is needed. Tachyphylaxis with the repeated use, and also, the potential for fluid retention and hyponatremia are additional issues to take into account [8].

Platelet Transfusions

Platelet transfusions often become necessary to address acute bleeding. In some cases, patients might develop antibodies against the GPIb protein. To mitigate this risk, it is now recommended to utilize specially selected platelet transfusions sourced from human leukocyte antigen (HLA)-matched single donors [9].

Cryoprecipitate

Cryoprecipitate contains high levels of vWF and fibrinogen and can be used as an adjunct in managing bleeding episodes in BSS. Cryoprecipitate is derived from pooled human plasma, leading to variability in its composition. The exact concentration of clotting factors may vary between batches, making it challenging to achieve consistent dosing. Cryoprecipitate also requires frozen storage and thawing before use. The need for timely preparation may lead to delays in emergency situations. Costs, limited availability, and the potential for allergic reactions are also issues to address [10].

The literature on perioperative and postoperative care for BSS patients remains scarce, primarily focusing on adult patients under 40 years old (Table 1) [11-20].

**Table 1:** Reports in the literature regarding the perioperative and postoperative care of patients with BSS DDAVP: 1-deamino-8-arginine-vasopressine; FFP: fresh frozen plasma; TEG: thromboelastogram; PLT: platelet; RBC: red blood cell; rFVIIa: recombinant factor VIIa; SC: subcutaneous; TXA: tranexamic acid; TID: three times a day

Author	Context	Preoperative care	Intraoperative care	Postoperative care
Kostopanagiotou et al., 2004 [11]	A 20-year-old woman for emergency laparotomy due to intra-abdominal bleeding	PLT, FFP, intranasal DDAVP 30 g, hydrocortisone 100 mg	RBC and FFP transfusions, TEG monitoring	None. The patient was discharged from the hospital on the fourth postoperative day.
Hacihanefioglu et al., 2007 [12]	A 30-year-old woman for dental extraction	rFVIIa (90 ug/kg) 2 hours prior to surgery	None	rFVIIa 2 h after surgery.
Tefre et al., 2009 [13]	A series of six surgical procedures in two brothers (30- and 23-year-old) with BSS included open-knee surgery, strabismus repair, dental extraction, right testis relocation, arthroscopic correction of the medial menisci	rFVIIa immediately before his operations with a dose of 96 to 104 ug/kg	None	rFVIIa starting at 2 h intervals after the procedure, increasing the interval between doses after the first 12 h (3/3h), 40 h (4/4h), 46 h (6/6h). A similar approach was adopted in the other surgical procedures. For the open-knee surgery repair was maintained for the first 124 h, and for the other procedures between 12 and 76 h after surgery.
Rodseth, 2010 [14]	A 48-year-old woman for elective repair of a supraumbilical hernia	Not mentioned	PLT, FFP, TXA (25 mg/kg) and DDAVP (0.3 µg/kg), TEG monitoring	RBCs and PLT; drains were removed on day 4 and the patient was discharged on day 6.
Bilal et al., 2010 [15]	A 68-year-old man for off-pump coronary artery bypass grafting	PLT in the morning of the surgery	TXA (2 g) as a bolus, followed by an infusion of 16 mg/kg/h	PLT prior to removal of the central venous and arterial lines. He was discharged on postoperative day 8.
Balci et al., 2014 [16]	A 9-year-old boy for tonsilloadenoidectomy and circumcision	Oral TXA (25 mg/kg per day), for 10 days prior to the procedure and PLT the day before	None	PLT during hospitalization and TXA for the first 10 days. The patient was discharged on the seventh postoperative day.
Bisland and Smith, 2014 [17]	A 40-year-old female for left total hip arthroplasty	TXA 1 g p.o. TID 24 hours prior to surgery and 20 mcg of DDAVP intravenously 1 hour prior to surgery	None	TXA 1 g p.o. TID continued until 14 days post-operatively; daily PLT up to post-operative day 14; DDAVP (20 mcg, SC) daily until post-operative day 4; the patient was discharged home on post-operative day 19.
Macêdo et al., 2015 [18]	A 28-year-old for an elective caesarean section	PLT on the course of three days prior to the caesarean section	None	None. The patient was discharged on day 7.
Ul Haq et al., 2015 [19]	A 20-year-old woman for an emergency caesarean section	PLT and 2 g of TXA before taking the patient to the operating room	None	PLT and 500 mg TXA every 6 h intravenously for 48 h, switched to oral regimen. The patient was discharged home uneventfully on the fourth postoperative day.
Boppana et al., 2018 [20]	A 17-year-old for posterior spinal fusion for idiopathic scoliosis	PLT	Prior to incision: pheresed platelet transfusion plus rFVIIa (1 mg). rFVIIa was repeated every 2-3 h intraoperatively. TXA (a loading dose of 50 mg/kg followed by a continuous infusion of 5 mg/kg/h during the procedure)	Pheresed platelet transfusion during the first postoperative day and α-amino caproic acid was administered intravenously and then per os for a total of 10 days.

We have presented a successful management case of a frail patient diagnosed with BSS undergoing a major surgical procedure. Employing a combined approach involving antifibrinolytics, recombinant factor, and platelet transfusions proved effective. Clinical protocols for anesthetic management during the perioperative period vary in pharmacological strategies and timing for clinical monitoring and hospitalization. However, there's a lack of specific management approaches tailored for elderly patients.

In our case, we opted for rigorous clinical monitoring in the PACU. We extended the pharmacological strategy by administering rFVIIa for the initial three days, along with platelet transfusions and tranexamic acid up until day 9. The occurrence of a hemorrhagic complication in the later postoperative phase emphasizes the necessity for robust perioperative planning and early establishment of a multidisciplinary perioperative team when dealing with BSS patients. It underscores the continuous need for comprehensive support and vigilant clinical monitoring due to the persistent bleeding risk associated with these patients.

## Conclusions

Our patient with BSS underwent a major surgical procedure and experienced hemorrhagic shock in the late postoperative period, which emphasizes the critical need for vigilant monitoring and tailored interventions in patients with coexisting bleeding disorders. Despite meticulous preoperative planning and transfusion support, unforeseen challenges emerged, stressing the importance of an interdisciplinary approach involving hematologists, surgeons, anesthesiologists and intensivists in managing complex cases.

The combination of antifibrinolytics, recombinant factor, and platelet transfusions proved effective in this scenario. Furthermore, this case serves as a reminder of the ongoing need for advancements in medical technology, such as novel hemostatic agents, improved platelet transfusion protocols, and enhanced intraoperative monitoring techniques, to optimize outcomes in patients with complex coagulopathies, undergoing surgical interventions.
